# Extract of *Rhus verniciflua* Bark Suppresses 2,4-Dinitrofluorobenzene-Induced Allergic Contact Dermatitis

**DOI:** 10.1155/2013/879696

**Published:** 2013-04-24

**Authors:** Dong Ki Park, Yang Gi Lee, Hye-Jin Park

**Affiliations:** ^1^Department of Life & Environmental Sciences, Konkuk University, 1 Hwayang-dong, Gwangjin-gu, Seoul 143-701, Republic of Korea; ^2^Cell Activation Research Institute, Konkuk University, 1 Hwayang-dong, Gwangjin-gu, Seoul 143-701, Republic of Korea; ^3^Ckadhc Jiangsu Lab, Suyu 1-dong, Gangbuk-gu, Seoul 142-874, Republic of Korea; ^4^Department of Bioscience and Biotechnology, Konkuk University, 1 Hwayang-dong, Gwangjin-gu, Seoul 143-701, Republic of Korea

## Abstract

*Rhus verniciflua* Stokes (RV) has traditionally been used as a food supplement and a traditional herbal medicine for centuries in Korea. Recent studies suggest that RV has potent antioxidative, antitumor, and anti-inflammatory properties. In this study, the anti-inflammatory effects of RV from mice sensitized with 2,4-dinitrofluorobenzene (DNFB) and activated macrophages were investigated. The results showed that RV reduced ear swelling and hyperplasia of ear tissue as well as an increase in vascular permeability, which are characteristics of allergic contact dermatitis (ACD) with evident histomorphological changes in epidermis and dermis. Decreased numbers of infiltrated mast cells were seen in RV extract treated group, using toluidine blue staining. RV extract significantly regulates the expression of inducible nitric oxide synthase (iNOS) at the translational level in activated macrophages. Furthermore, RV extract and its active compound, fisetin, attenuated the level of tumor necrosis factor-**α** (TNF-**α**) and interleukin 6 (IL-6) mRNA in LPS-stimulated macrophages. Anti-ACD effect of RV extract may be due to the suppression of iNOS and proinflammatory cytokines which might be mediated via the NF**κ**B signaling pathways. Collectively, RV extract has potential for alleviating ACD-like symptoms induced by DNFB in the mouse.

## 1. Introduction

Allergic contact dermatitis (ACD) is a highly prevalent skin inflammatory disease and is characterized by allergic signs and symptoms of the skin, such as redness, edema, warmth, and pruritus, accompanied with scaling and dryness [1]. ACD has two distinct phases, that is, sensitization and challenge phases. The sensitization phase results in the induction of hapten-specific CD8^+^ and CD4^+^ T cells and is considered to have no clinical consequences. The challenge phase of CD occurs when sensitized individual is exposed to the same hapten, which recruits effector T cells and triggers the inflammatory process [2]. Study of the pathophysiology of ACD is derived mainly from animal models in which the skin inflammation is caused by repeated topical application to the haptens. Among these haptens, 2,4-dinitrofluorobenzene (DNFB) is known to mediate allergic contact dermatitis by CD8^+^ cytotoxic T cells and mast cells and thus is used to produce skin contact hypersensitivity in mice models [3]. Repeated topical application of DNFB triggers a hypersensitive reaction and induces a shift in the cutaneous cytokine milieu from a Th1 to a Th2 profile [4, 5], augmenting immune cell recruitment, or allergic inflammation. Macrophages are a major subpopulation of cells in the murine dermis, and it has been well documented that RAW264.7 cells respond to DNFB [6]. Numerous lines of evidence describe that activated macrophages play a crucial role in inflammatory process through producing proinflammatory cytokines and mediators.

*Rhus verniciflua* Stokes (RV) has been traditionally used as medicine agents for antiviral, cathartic, diaphoretic, anti-rheumatic, and sedative activities in East Asia [7]. Recently RV is reported to contain strong antioxidant, antitumor properties and anti-inflammatory activities [8–10]. These activities of RV are presumably due to the presence of polyphenolic compounds as fisetin and quercetin [10, 11]. Recently, Rhus products have been commercialized due to a national project of the Ministry of Agriculture and Forestry of Korea, and it is required to detoxify Rhus poison such as urushiol [12]. Based on this background, we investigated anti-inflammatory activity of RV in which allergenic urushiol was removed, using a mouse model of DNFB-induced allergic CD and the mechanism involved.

## 2. Methods

### 2.1. Preparation of RV Extract

Bark of *Rhus verniciflua* Stokes was provided by Ckadhc Jijangsu lab in Seoul, South Korea, and was authenticated by The Korean Food & Drug Administration (KFDA). The allergenic urushiol from the RV was removed by previously described procedures [13]. The detoxification was verified by KFDA (no. 204074). A voucher specimen (Kucari1401) was deposited at herbatorium of Konkuk University. Four hundred grams of RV bark was crushed. The extract was then dried in an oven at 65°C and ground. This powder was then boiled with water at 100°C. This extract was filtered with Whatman paper 4 (Whatman, Kent, UK), and the residual water was removed by vacuum/dry evaporation. The yield was 6.993% (w/w) (Supplementary Figure 1 available online at http://dx.doi.org/10.1155/2013/879696).

### 2.2. Experimental Animals

Six-week-old female C57BL6/N mice (6-week-old) were purchased from Orient Bio (Eumsung, Korea). Mice were housed in a controlled barrier facility at temperature (23 ± 2°C), humidity (35–60%), and photoperiod (12 h light: 12 h darkness cycle) were kept constantly. Experiments were performed in accordance with the institutional guidelines (The Institutional Animal Care and Use Committee (IACUC) at Konkuk University (Seoul, Korea)). Also, all animal care was in accordance with standards set seventh in the Guide for the Care and Use of Laboratory Animals. The protocol ku10009 was approved by Konkuk University Medical center IACUC for this study.

### 2.3. Induction of Allergic Contact Dermatitis (ACD) by 2, 4-Dinitrofluorobenzene (DNFB)

CD was induced by repeated treatment of DNFB solution to each surface of right ear and shaved abdomen in C57BL/6N mice (*n* = 5). 100 *μ*L of 0.5% DNFB in acetone/olive oil (4 : 1. v/v) was painted on the shaved abdomen skin on days 1, 2, 3, 5, and 7. After painting, mice were challenged by 0.2% DNFB (25 *μ*L) painting to the ear on day 9. Control mice were treated with acetone/olive oil (4 : 1. v/v) in the absence of DNFB. RV (500 mg/kg) was orally administrated once a day for eleven days.

### 2.4. Histological Study

The fixed ear tissue was embedded in paraffin blocks and then sectioned at 5 *μ*m thickness. Sections were stained with hematoxylin (BBC Biochemical, Mount Vernon, Washington, USA) and eosin (Shimakyu's Pure Chemicals, Osaka, Japan) (H&E) and then observed using a light microscope. Digital photomicrographs were taken from representative areas at a fixed magnification of 200x.

### 2.5. Measurement of the Ear Thickness

The thickness of ear, stained with hematoxylin and eosin, was measured using Axiovision image analysis 4.7 software (Carl Zeiss Vision GmbH, Munich, Germany). Stained tissue images were captured with the digital microscope camera and their thickness was measured.

### 2.6. *In Vivo* Permeability Assay

A 1% solution of Evans blue dye (Sigma-Aldrich) was injected into the tail vein of mice. After one hour, mice were sacrificed. Evans blue dye was then extracted from tissues in formamide (Sigma-Aldrich, St. Louis, MO, USA) at 60°C overnight. After measurement of optical density at 600 nm in a spectrophotometer, the concentration of Evans blue dye (ng/mg) was calculated based on a standard curve of known amounts of Evans blue dye.

### 2.7. Toluidine Blue Stain

Sections were stained with toluidine blue (Sigma-Aldrich, St. Louis, MO, USA) for mast cells. After deparaffinization and hydration, sections were stained with toluidine blue solution for 2-3 minutes and dehydrated in alcohol and cleared in xylene. Sections were observed under the light microscope, and the number of mast cells per field of view (×400) was determined.

### 2.8. Immunohistochemistry

To assess NF-*κ*B expression in the ear tissues, the Immunocruz staining system kit (Santa Cruz Biotechnology, California, USA) was used. 5 *μ*m thick tissue sections were deparaffinized with xylene and rehydrated with graded ethanol solutions. The sections were incubated with a rabbit antibody against NF-*κ*B p65 (Santa cruz Biotechnology, CA, USA, 1 : 600) for 1 hour. After washing, the sections were incubated with a biotinylated secondary antibody for 30 min and an HRP-streptavidin complex to detect secondary antibody for 30 min. The sections were developed by DAB chromogen kit (Vector laboratories, Burlingame, California, USA). The sections were counterstained with 1% methyl green for 1 min.

### 2.9. Cell Culture

RAW264.7 macrophages (TIB-71) cells were obtained from the American Type Culture Collection (Manassas, Virginia, USA) and cultured in DMEM (Invitrogen Co., Carlsbad, CA, USA) supplemented 1% penicillin and 10% FBS (Gibco BRL, Grand Island, NY, USA). Cells were grown at 37°C with 5% CO_2_ in a humidified incubator.

### 2.10. Cell Viability

The viability of RAW264.7 cells was determined by the EZ-CyTox kit (Daelillab Service CO, Korea) as described previously [14]. RAW264.7 cells (2 × 10^4^ cells/well) were plated on a 96-well plate and incubated in the presence or absence of RV extracts at concentrations of 25, 50, 100, and 200 *μ*g/mL for 24 h, respectively. A fixed amount (10 *μ*L) of EZ-CyTox reagent was added to each well. After incubation for 2 h at 37°C, absorbance at 450 nm was detected by using an ELISA Multi-Detection Reader (Tecan, Mannedorf, Switzerland).

### 2.11. Measurement of NO

Nitrite concentrations, an indicator of NO production, in RAW264.7 cell culture media were measured as described previously.

### 2.12. Immunoblot Analysis

Immunoblotting analysis was performed as described previously [14, 15]. Total protein (20 *μ*g) was separated from each sample by electrophoresis on a 12% SDS-PAGE polyacrylamide gel and electrophoretically transferred onto polyvinylidene fluoride membranes (PVDF) (Bio-Rad Laboratories, Berkeley, California). The membranes were incubated with 5% skim milk solution followed by incubation with primary antibody. The membranes were washed in a 1x PBS-T buffer and incubated with HRP-conjugated secondary antibodies (Santa Cruz, CA, USA, 1 : 5,000) for 1-2 h. The immunoreactive bands were detected using the enhanced chemiluminescence Western blotting detection system (Biosesing, Seoul, Korea) (iNOS, *β*-Actin, Lamin B, NF*κ*B p65 (Santa Cruz Biotechnology, CA, USA), COX2, and I*κ*B*α* (Cell Signaling Technology Inc., Danvers, MA, USA)).

### 2.13. RNA Extraction, cDNA Synthesis, and Real-Time Polymerase Chain Reaction (Real-Time PCR)

Total RNA of RAW264.7 cells was extracted using a total RNA isolation kit (Macherey-Nagel GmbH & Co., Düren, Germany). Total RNA was converted to cDNA using a cDNA synthesis kit (*Fermentas*, St. Leon-Roth, Germany). Amplification reactions were carried out in a total volume (20 *μ*L) of 10 *μ*L containing 2x SYBR Green PCR Master Mix, 2 *μ*L 10x QuantiTect Primer Assay (Qiagen, Valencia, CA, USA), 100 ng cDNA and 6 *μ*L RNase-free water. Real-time PCR was performed using an ABI500 thermal cycler (Applied Biosystems, Foster City, CA, USA). Data were normalized for the amount of glyceraldehydes-3-phosphate dehydrogenase mRNA. Levels of iNOS, IL-6, and TNF-*α* mRNAs mRNAs were measured by ΔΔC_T_ value real-time PCR. Specific primer sets for iNOS (QT00100275), IL-6(QT00098875), TNF-*α* QT00104006), and GAPDH (QT01658692) were designed using the Primer Express program (Applied Biosystems, CA, USA).

### 2.14. HPLC Analysis

In order to analyze the compounds in the extracts, high performance liquid chromatography (HPLC) experiments were carried out on an Agilent 1260 Infinity HPLC system (Santa Clara, CA, USA) with the reversed phase column (Luna C18, 250 × 4.6 mm, 5 *μ*m diameter, Phenomenex, Torrance, CA, USA). The flow rate and injection volume were 1.2 mL/min and 5–20 *μ*L, respectively. The chromatograms were detected at 260 nm and collected at 30°C. Fisetin was purchased from Sigma-Aldrich (St. Louis, MO, USA) and used without further purification. One mg of adenosine was dissolved in 1 mL of 50% ethanol and filtrated using 0.45 *μ*m membrane filters. While the mobile phase for the ethanol extract of RV was 8% aqueous methanol, that for the fractions separated based on the hydrophobicity was 6% aqueous methanol containing 0.1% KH_2_PO_4_.

### 2.15. Statistical Analysis

Results are expressed as mean ± S.E. (*n* = 5 per group). Student's *t*-test or one-way ANOVA/Dunnett's *t*-test was used for assessing significance between control group and sample treated groups. Statistical analysis was performed using SPSS, version 12 (SPSS Inc., Chicago, IL, USA).

## 3. Results

### 3.1. Effect of RV Extract on the Vascular Permeability and Ear Swelling in DNFB-Induced Allergic Contact Dermatitis (ACD) Mice

**Figure 1:**
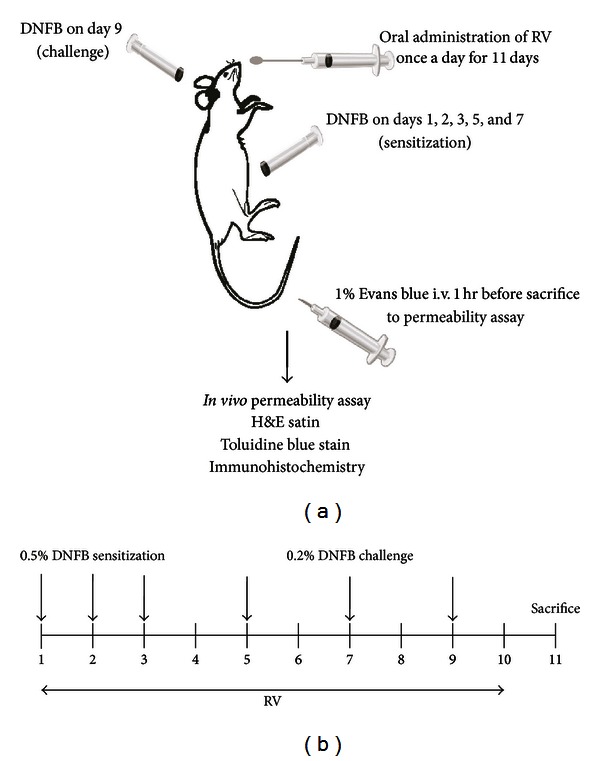
Scheme of the experimental design.

Accumulating studies have reported pharmacological effects of RV extract. In order to explore a therapeutic role of RV extract in skin inflammatory diseases *in vivo*, we employed a mouse model of allergic contact dermatitis. To assess the anti-ACD activity of RV extract, Evans blue was administered intravenously (i.v.) to anaesthetized animals. During all experiments, Evans blue was administered 1 h before the animals were sacrificed. Repeated topical application of 2,4-dinitrofluorobenzene (DNFB) caused Evans blue leakage in ears. Quantification of the extravasated Evans blue dye in the ear tissue provides a degree of vascular permeability changes, which reflects ear swelling [16]. Evans blue leakage in the ear tissue was significantly decreased in RV extract treated group, compared to DNFB-treated group (*P* < 0.01) (Figures 1(a) and 1(b)). The ear thickness of the RV treated group was 0.273 ± 0.006 mm while that of DNFB-treated group was 0.437 ± 0.007 mm (*P* < 0.05) (Figure 1(c)). Our data suggests that RV extract significantly reduces the ACD symptoms.

### 3.2. Effects of RV Extract on Histopathological Changes of Ear Tissues in DNFB-Induced Allergic Contact Dermatitis Mice

DNFB-induced ACD caused severe inflammation in ear tissues showing a typical dermatopathological appearance of ACD, namely, hyperkeratosis, and spongiosis together with marked edema in the epidermis and dermis associated with a massive infiltration of inflammatory cells [17]. For histopathological analysis, we excised the ear on day 11 after challenging with DNFB and stained it with hematoxylin-eosin. The epidermis and dermis in the DNFB-treated mice displayed strong edema, hyperplasia, and spongiosis as well as an increase in the infiltration of inflammatory cells. A notable reduction in inflammatory cells in epidermis and dermis and hyperplasia was seen in RV extract treated group (Figure 3(a)). The epidermis of the ear tissue from RV extract treated group showed a 1.4-fold decrease in thickness as compared to that of DNFB-treated group (Figure 3(b)). Consistent with the decreased ear swelling, RV extract treated group showed a reduced thickness in epidermal and dermal ear tissues, compared to DNFB-treated group. This result suggests that the reduction of epidermal tissue thickness by RV extract may be associated with its anti-inflammatory activity.

### 3.3. Effect of RV Extracts on Mast Cell Infiltration in DNFB-Induced Allergic Contact Dermatitis Mice

Mast cells are one of the major infiltrated inflammatory cells in the skin area of allergic contact dermatitis (ACD) patients. These cells are activated by allergen sensitized IgE through their cell surface high-affinity IgE receptor, subsequently secreting granular mediators, such as prostaglandin, leukotrienes, and various proinflammatory cytokines [18]. Thereby, we assessed the effect of RV extract on the infiltration and degranulation of mast cells, using toluidine blue staining. The numbers of infiltrating and degranulated mast cells were significantly reduced in the upper dermis of RV extract treated group when compared to that of the DNFB-treated group (Figure 4).

### 3.4. Effects of RV Extract on NO and iNOS in LPS-Treated RAW264.7 Cells

Macrophages, a subpopulation of cells in the dermis, are a useful therapeutic target for ACD [19]. It is reported that ACD significantly induces the levels of proinflammatory mediators, such as NO in monocytes and macrophages. LPS induces RAW 264.7 cell activation with a change in morphology during inflammatory responses [20]. Since RV extract reduced the symptoms of contact dermatitis (CD) in theanimal model, we further investigated whether RV extract could suppress the production of proinflammatory mediators such as nitrite in LPS-stimulated RAW 264.7 macrophage cell line. The results showed that LPS induced significant increases of NO and iNOS production in RAW264.7 cells, compared with the control (Figure 5). RV extract attenuated NO release and iNOS protein expression induced by LPS treatment in a dose-dependent manner (Figure 5). In addition, RV extract did not influence cell viability as evaluated by CCK-8 assay (Figure 5), indicating that its inhibitory effects were unrelated to a direct cytotoxicity [21].

### 3.5. Effects of RV Extract on Proinflammatory Cytokines in LPS-Treated RAW264.7 Cells

We tested whether RV treatment could reduce TNF-*α* production upon LPS stimulation. RV extract significantly decreased TNF-*α* mRNA levels, compared to the control (Figure 6(a)). Together with TNF-*α*, interleukin (IL)-6 plays a pivotal role in skin inflammation (e.g., contact dermatitis) and hypersensitivity response [22]. RV extract inhibited IL-6 mRNA compared to LPS alone (*P* < 0.001) (Figure 6(b)). Collectively, these data indicate that RV extract may have an anti-inflammatory effect through inhibiting the productions of proinflammatory cytokines and mediators.

### 3.6. Effects of RV Extract on NF-*κ*B Translocation

To further elucidate the mechanism underlying the inhibitory effect of RV extract on macrophage activation and ACD, we investigated whether RV extract could influence the LPS-induced activation of NF-*κ*B, using western blot analysis. NF-*κ*B activation is crucial for the inflammatory response in RAW264.7 cells. The hyperphosphorylation of I*κ*B and its subsequent degradation is an essential step in NF-*κ*B activation by various stimuli [23]. Here, we separated the nuclear proteins and cytosolic proteins. Equivalent proteins from nucleus were used to evaluate whether the NF-*κ*B translocation was affected by RV and the cytosolic proteins were used to check the degradation of I*κ*B. We found that RV extract strongly inhibited NF-*κ*B p65 subunit translocation (Figure 7(a)) as well as the I*κ*B degradation (Figure 7(b)). In concert with the inhibition of NF-*κ*B p65 subunit translocation, RV extract significantly reduced I*κ*B-*α* phosphorylation. These findings were supported by immunohistochemistry detection of the intracellular NF-*κ*B p65 subunit in the ear tissue of DNFB-induced allergic contact dermatitis (Figure 7(c)). Low level of NF-*κ*B p65 was observed in the control and RV-extract treated group, whereas there was a striking elevation in immunoreactivity for NF*κ*B in the tissue of the DNFB-treated mice. These data indicate that RV extract alleviates ACD and macrophage activation through the suppression of NF-*κ*B function.

### 3.7. HPLC Analysis of Components in RV Extract and Its Anti-Inflammatory Activity

Many studies demonstrate that flavonoids such as fisetin and quercetin are the major active components of RV, possessing anti-inflammatory activity [8]. Among them, fisetin was analyzed in the RV extract, whose urushiol was removed, by HPLC analysis. In the HPLC experimental condition for the extract of RV, the authentic fisetin was observed at the retention time of 18 min in its chromatogram. The concentration of RV was prepared as 17.4 mg/mL (Figure 8(a)). Fisetin was observed from RV and its concentration was 0.391%. Fisetin significantly reduced the LPS-stimulated production of proinflammatory cytokine mRNAs such as TNF-*α* and IL-6 as well as iNOS mRNAs in LPS-stimulated RAW264.7 cells (Figure 8(b)).

## 4. Discussion

Currently, the best treatment for allergic contact dermatitis (ACD) is to avoid the offending agents, such as eliminating the contact allergen. Topical treatments such as corticosteroids and phototherapy are used to alleviate this symptom but their effects are temporary with undesirable side effects. Recently, anti-inflammatory agents are considered to be alternative treatment options for treating ACD. Increased attention has been paid to medicinal plants with anti-inflammatory due to their efficacy and safety. For instance, RV has been traditionally used to treat inflammatory diseases and gastrointestinal problems and to improve health condition in East Asia. Additionally, it is reported that RV has a wide range of effects including antioxidant, antiproliferative activity and anti-inflammatory effects [24]. However, some literatures demonstrate that RV caused contact dermatitis due to the presence of urushiol. Based on this background, we examined the anti-inflammatory effects and its mechanism of actions of RV extract, in which allergenic urushiol was removed, using an animal model of allergic contact dermatitis (ACD), and RAW264.7 macrophage cells.

**Figure 2:**
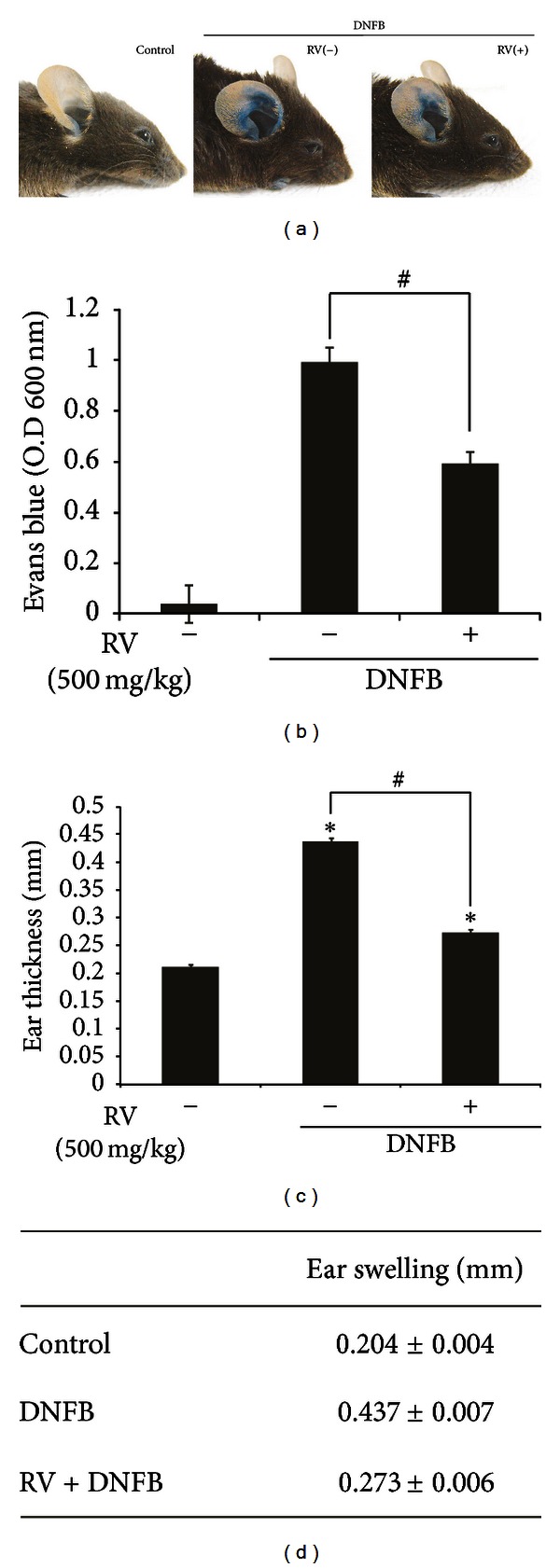
Effect of RV extract on the ear swelling and vascular permeability in DNFB-induced allergic contact dermatitis mice. (a) Representative photographs were shown. (b) The amount of extracted Evans blue dye from each ear was determined with a spectrophotometer at OD 600 nm. Each value represents the mean ± standard error (S.E.) of three independent experiments (*n* = 5 per group) (*P < 0.01 versus DNFB). (c) Thickness of H&E stained ear tissue was measured with Axiovision image analysis 4.7 software. Data are expressed as mean ± standard error (S.E.) of three independent experiments (*n* = 5 per group) (*P < 0.001 versus control; ^#^ *P* < 0.001 versus DNFB).

**Figure 3:**
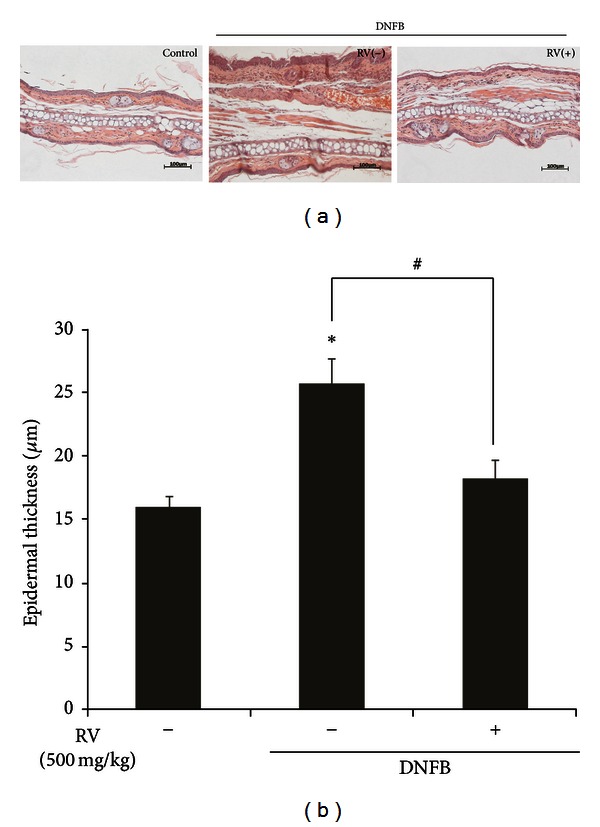
Effects of RV extract on epidermal thickness and histopathological changes in DNFB-induced allergic contact dermatitis mice. (a) Histological changes were determined by H&E staining. Scale bar = 100 *μ*m. Original magnification, 200x. (b) Epidermal thickness of the H&E stained ear tissue was measured using Axiovision image analysis 4.7 software. Data are expressed as mean ± standard error (S.E.) of three independent experiments (*n* = 5 per group) (*P < 0.001 versus control; ^#^ *P* < 0.05 versus DNFB).

**Figure 4:**
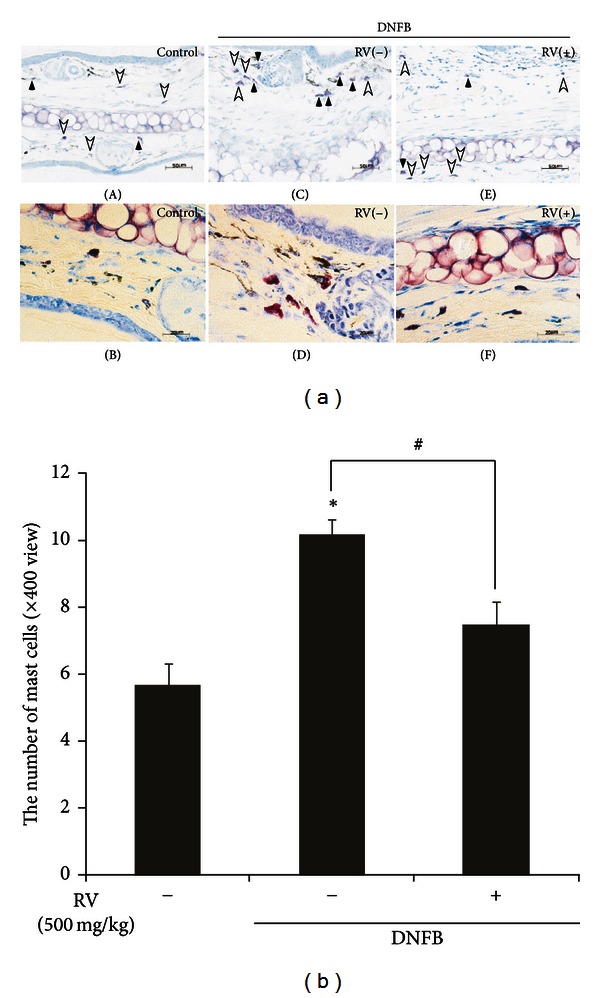
Effect of RV extract on the mast cell infiltration in DNFB-induced allergic contact dermatitis mice. (a) Toluidine blue-stained ear sections. White arrowhead and black arrowhead indicate granulated mast cells and degranulated mast cells, respectively. Scale bar = 50 *μ*m (top panel); scale bar = 20 *μ*m (bottom panel). (b) Quantitative analysis of the number of mast cells. Numbers of mast cells are presented as means ± standard error (S.E.) of three independent experiments (*n* = 5 per group) (^a^ *P* < 0.001 versus control; ^b^ *P* < 0.01 versus DNFB).

The repeated topical application of 2,4-dinitrofluorobenzene (DNFB) causes a contact hypersensitivity reaction. Sensitization of dorsal skin with DNFB and subsequent challenge of the ear resulted in ear swelling, hyperplasia, and increased vascular permeability (as measured by the extravasation of Evans blue dye) within 24 h. Oral administration of RV reduced erythema and edema as well as decreased Evans blue leakage (vascular permeability) that was induced by DNFB (Figure 2). Furthermore, the inhibition of infiltration of mast cells strongly suggests that RV is a promising pharmacological candidate for skin disorders, such as ACD or atopic dermatitis.

**Figure 5:**
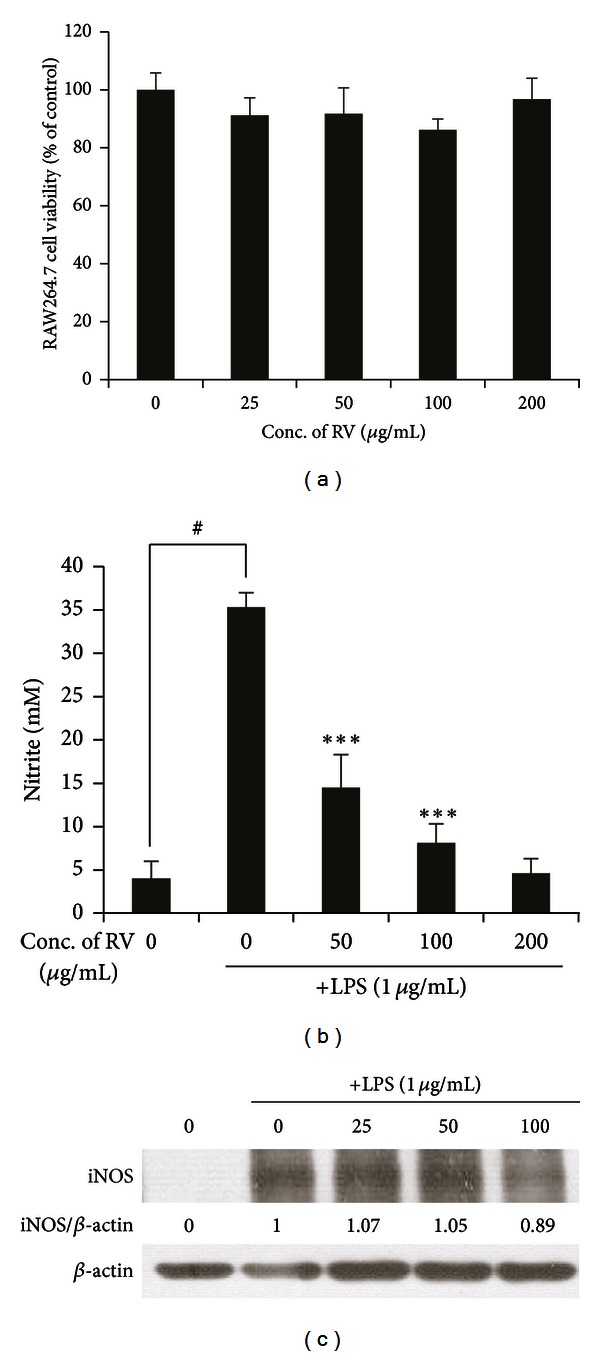
Effect of RV extract on NO production and iNOS protein expression in lipopolysaccharide- (LPS-) stimulated RAW264.7 macrophages. (a) Cell viability of RV extract was determined by the EZ-CyTox kit. (b) Nitrite production in medium was determined using Griess reagent assay. (c) The protein expression of iNOS was analyzed by Western blot analysis. Data are represented as mean ± standard error (S.E.) of three independent experiments. One-way ANOVA was used for comparisons of multiple group means followed by Dunnett's *t*-test (^#^ *P* < 0.001 versus control; *P < 0.01, **P < 0.05, ***P < 0.001 versus LPS-stimulated control).

To address its potential mechanism of anti-ACD action, we performed *in vitro* cell culture studies by testing whether RV extract could modulate the effector function of macrophages. Macrophages gained attention as a useful therapeutic target for contact allergies [19]. Nitrosative stress from inducible-NOS-(iNOS-) derived NO contributes to the progression and the pathogenesis of ACD [25]. We found that RV extract significantly reduced the nitrite production and inhibited the expression of inducible nitric oxide synthase (iNOS) at protein levels in LPS-stimulated RAW264.7 cells (Figure 5). These results suggest that RV may suppress macrophage activation, which can lead to allergic inflammation.

**Figure 6:**
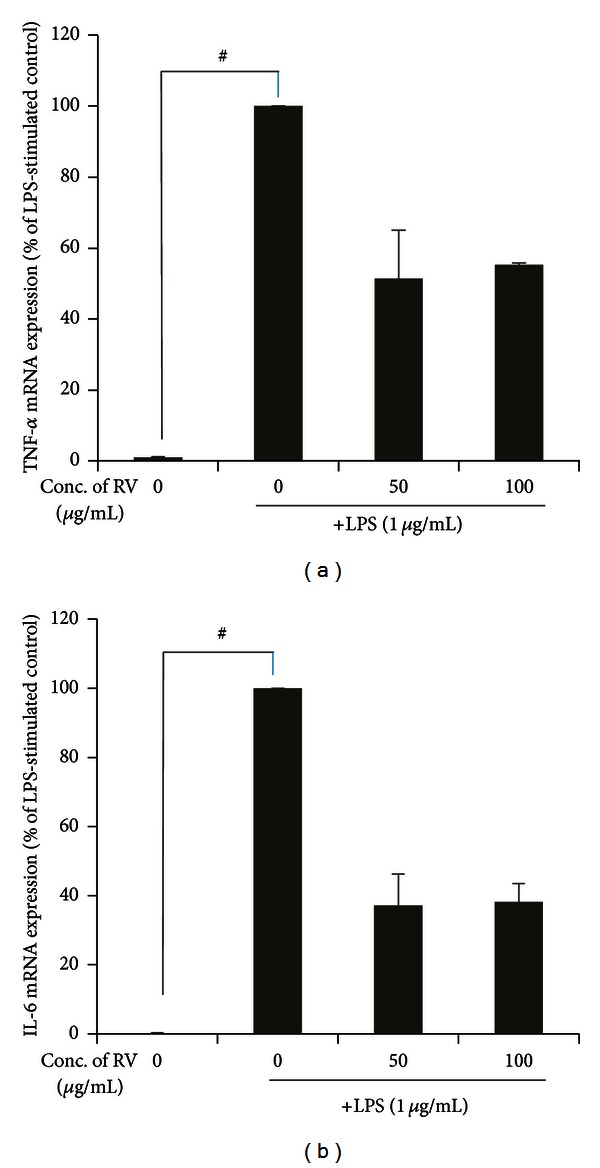
Effect of RV extract on the level of TNF-*α* and IL-6 mRNAs in LPS-stimulated RAW264.7 macrophages. (a-b) Cells were incubated for 5 h with 1 *μ*g/mL of LPS in the absence or presence of RV extract. RV extract was added 1 h before the incubation with LPS. Levels of TNF-*α* and IL-6 mRNA in LPS-stimulated RAW264.7 cells were analyzed by real-time PCR and determined by quantitative ΔΔC_T_ real-time PCR, using GAPDH mRNA as the internal control. Data are represented as mean ± standard error (S.E.) of three independent experiments. One-way ANOVA was used for comparisons of multiple group means followed by Dunnett's *t*-test (^#^ *P* < 0.001 versus control; *P < 0.01, **P < 0.05, ***P < 0.001 versus LPS-stimulated control).

**Figure 7:**
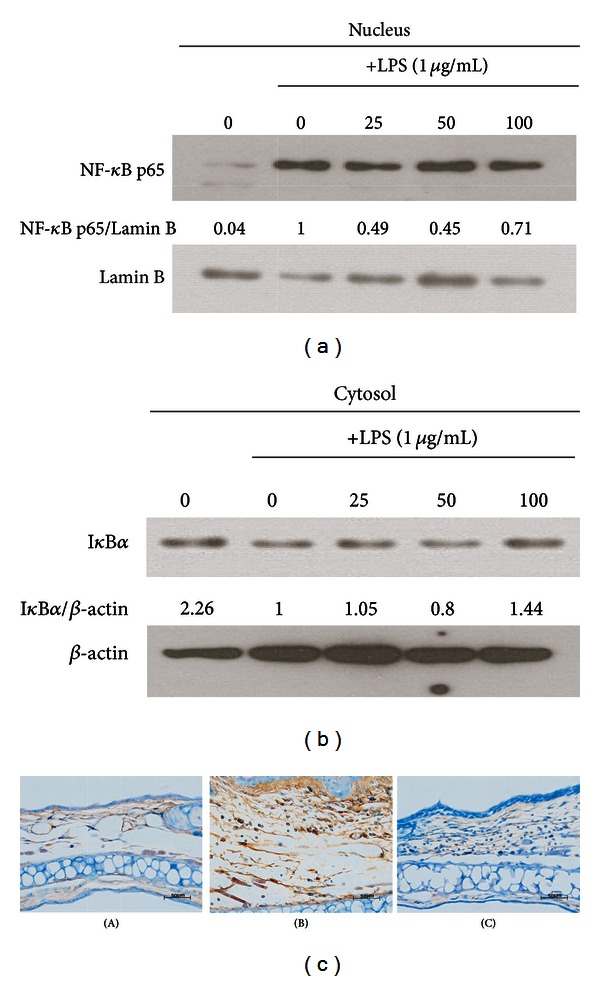
Effects of RV extract on the I*κ*-B and NF-*κ*B in LPS-stimulated RAW264.7 macrophages. (a, b) Cells were incubated for 30 min with 1 *μ*g/mL of LPS in the absence or presence of RV extract. RV extract was added 1 h before the incubation with LPS. The protein expression of I*κ*-B and NF-*κ*B was analyzed by Western blot analysis and quantified by densitometric analysis. (c) The immunohistochemical analysis was used to monitor the protein expression of NF*κ*B in the ear sections. Scale bar = 50 *μ*m. Original magnification, 400x. (A) Control, (B) RV(−), (C) RV(+). Data are represented as mean ± standard error (S.E.) of three independent experiments. One-way ANOVA was used for comparisons of multiple group means followed by Dunnett's *t*-test (^#^ *P* < 0.001 versus control; *P < 0.01, **P < 0.05, ***P < 0.001 versus LPS-stimulated control).

LPS treatment induced morphological changes and significantly increased production of proinflammatory cytokines, including TNF-*α* and IL-6 in RAW 267.4 cells (Figure 6). TNF-*α* plays a major role in inflammatory and autoimmune disorders [20], that is produced mainly by T cells and macrophages in response to a variety of stimulations. Mouse defective in TNF-*α* production shows resistance to ACD [26]. IL-6 is a proinflammatory cytokine that plays an important role in the acute phase response of ACD [21–27]. These cytokines are important components of innate and adaptive immunity that mediate activation and trafficking of various immune cells in response to infection or injury. RV inhibited LPS-induced production of TNF-*α* and IL-6 mRNAs (Figure 6).

To elucidate the molecular mechanism underlying the ability of RV to suppress ACD, we examined whether RV might be associated with the inhibition of NF-*κ*B-dependent inflammation, which are the main signaling pathways that lead to the transcription of most of the proinflammatory genes such as TNF-*α* and IL-6 [28, 29]. Our *in vitro* studies suggested that NF-*κ*B blockade by RV extract suppressed the macrophage activation. In concert with *in vitro* studies, RV extract attenuated NF-*κ*B expression in the ear tissue of DNFB-induced allergic contact dermatitis. These findings implied that RV extract might attenuate many inflammatory responses, including ACD by inhibiting the NF-*κ*B translocation.

**Figure 8:**
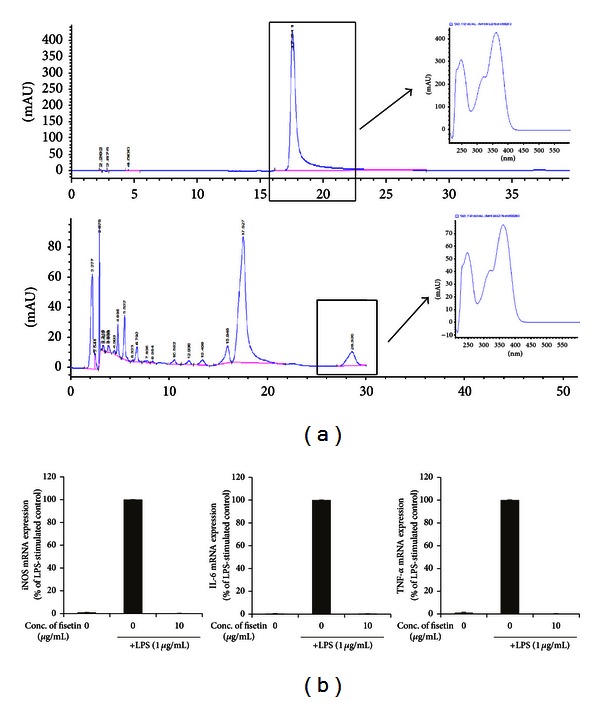
Analysis of the phytochemical profile of RV extract and the inhibitory activity of fisetin on TNF-*α*, IL-6, and iNOS mRNA expression in LPS-treated RAW264.7 cells. (a) Phytochemical characteristics of RV extract were analyzed by high performance liquid chromatography (HPLC). (b) LPS-induced TNF-*α*, IL-6, and iNOS mRNA expression in fisetin-treated RAW264.7 cells was examined by quantitative ΔΔC_T_ real-time PCR. One-way ANOVA was used for comparisons of multiple group means followed by Dunnett's *t*-test (^#^ *P* < 0.001 versus control; *P < 0.01, **P < 0.05, ***P < 0.001 versus LPS stimulated control).

Many studies demonstrate that flavonoids, including fisetin and quercetin, are the major active components of RV [10]. HPLC analysis data suggest that RV extract contains fisetin. It was reported that fisetin possessed anti-inflammatory [10], anticancer [30], and antioxidative activities [31]. Fisetin is known to suppress the production of tumor necrosis factor-*α* (TNF-*α*), IL-6, IL-8, and monocyte chemotactic protein-1 (MCP-1) in a rheumatoid arthritis model [32]. Consistent with previous reports, fisetin inhibited the level of TNF-*α* and IL-6 mRNAs in LPS-stimulated RAW264.7 cells (Figure 8). Anti-ACD activity of RV extract might be due to the presence of fisetin. Currently, we are studying the anti-ACD activity of fisetin (paper in preparation). However, we cannot rule out the possibility that other bioactive compounds in RV extract contribute to its anti-ACD activity.

In conclusion, we demonstrate that RV extract suppresses ACD symptoms such as ear swelling, hyperplasia, and vascular permeability in the ear tissue after exposure to the contact allergen DNFB. RV inhibited the production of proinflammatory mediators and proinflammatory cytokines in LPS-stimulated RAW264.7 cells through blocking NF-*κ*B translocation. This result implies RV exerts anti-inflammatory activity by regulating inflammatory responses mediated by NF-*κ*B pathway. Among active components of RV extract, fisetin was found to be particularly effective in inhibiting the inflammation parameters in activated macrophages. These findings enhance our understanding of the effects of RV extract on ACD. Here, we verified that RV had a therapeutic advantage on DNFB-induced allergic contact dermatitis, which might be related to its anti-inflammatory activity.

## Supplementary Material

The extraction procedure of RV is briefly described in the Supplementary Figure 1.
