# Supplementary figures and images for: Extract of Rhus verniciflua Bark Suppresses 2,4-Dinitrofluorobenzene-Induced Allergic Contact Dermatitis
Source: Evid Based Complement Alternat Med. 2013 Apr 24;2013:879696. doi: 10.1155/2013/879696 (PMC3655595; doi:10.1155/2013/879696)

## Supplementary Figure 1

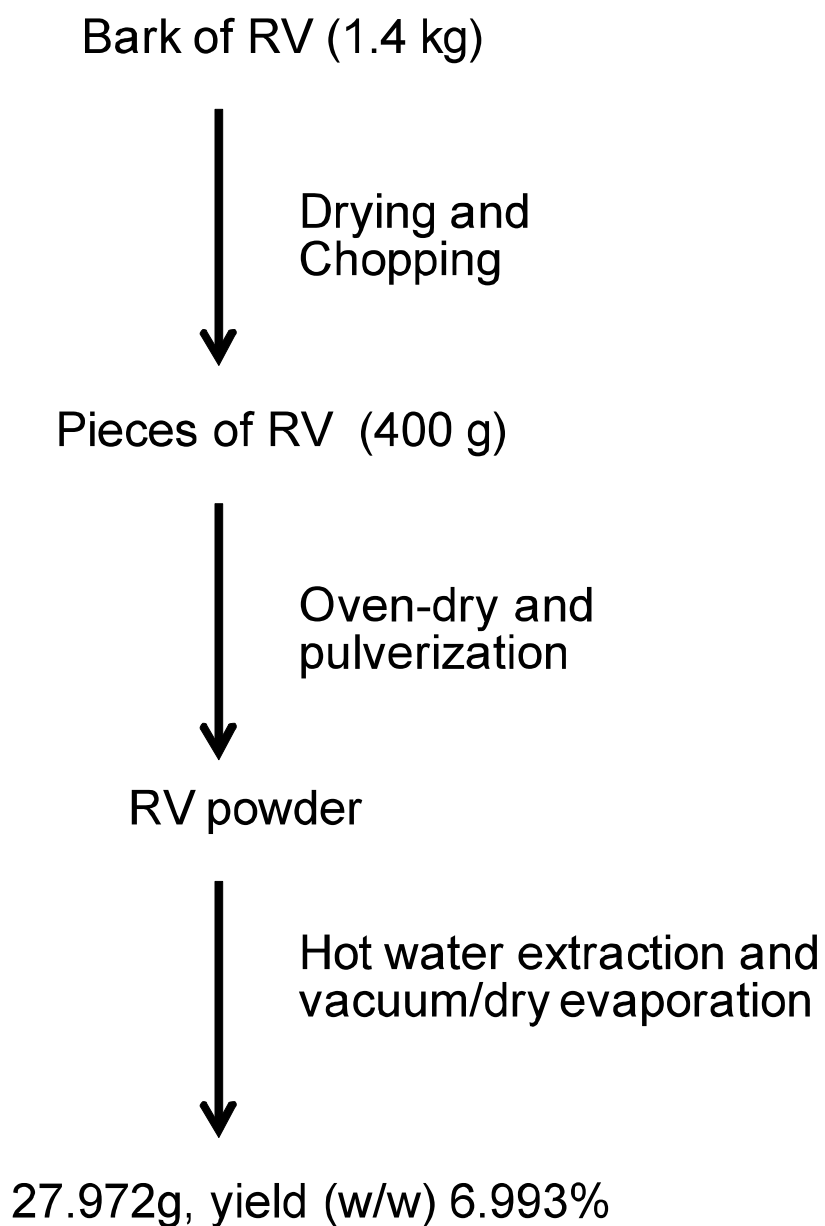

Supplement: Supplementary file 1 — The extraction procedure of RV is briefly described in the Supplementary Figure 1. [file 879696.f1.pdf]
